# New Developments in Gastric Neuroendocrine Neoplasms

**DOI:** 10.1007/s11912-021-01175-y

**Published:** 2022-01-20

**Authors:** Klaire Exarchou, Nathan A. Stephens, Andrew R. Moore, Nathan R. Howes, D. Mark Pritchard

**Affiliations:** 1 Department of Molecular and Clinical Cancer Medicine, Institute of Systems, Molecular and Integrative Biology, University of Liverpool, The Henry Wellcome Laboratory, Nuffield Building, Crown Street, Liverpool, L69 3GE UK; 2 Department of Upper Gastrointestinal Surgery, Liverpool University Hospitals NHS Foundation Trust, Liverpool, UK; 3 Department of Gastroenterology, Liverpool University Hospitals NHS Foundation Trust, Liverpool, UK

**Keywords:** Gastrin, Neuroendocrine tumour, Carcinoid, Enterochromaffin-like (ECL)-cell, stomach, Atrophic gastritis

## Abstract

**Purpose of Review:**

Gastric neuroendocrine neoplasms (g-NENs) are a rare type of stomach cancer. The three main subtypes have different pathogeneses, biological behaviours and clinical characteristics, so they require different management strategies. This article will provide an overview of g-NENs and highlight recent advances in the field.

**Recent Findings:**

Molecular profiling has revealed differences between indolent and aggressive g-NENs, as well as a new somatic mutation responsible for some familial type I g-NENs. Novel biomarkers have been developed which will hopefully improve diagnosis, treatment, risk stratification and follow-up. Patient treatment is also changing, as evidence supports the use of less aggressive options (e.g. endoscopic surveillance or resection) in some patients with more indolent tumours.

**Summary:**

g-NEN heterogeneity poses challenges in understanding and managing this rare disease. More basic science research is needed to investigate molecular pathogenesis, and future larger clinical studies will hopefully also further improve treatment and patient outcomes.

## Introduction

Neuroendocrine neoplasms (NENs) are heterogeneous tumours which have diverse clinical and biological characteristics. They arise from secretory cells of the diffuse neuroendocrine system, can occur at almost any anatomical site and share major molecular morphological and protein expression signatures as well as site-specific markers. Overall, NENs are subdivided into well-differentiated neuroendocrine tumours (NETs), poorly differentiated neuroendocrine carcinomas (NECs) and mixed neuroendocrine-non-neuroendocrine neoplasms (MiNENs), in which both components are substantial and account for at least 30% of the total neoplasm [1]. NEN classification is based on the type of tumour differentiation and the extent of tumour proliferation (grade) as determined by mitotic index and Ki-67 immunohistochemistry (Table 1). An increased risk of angioinvasion and metastatic potential is seen in higher grade and more poorly differentiated tumours, and, if disseminated, such NENs have a poorer prognosis.

**Table 1:** World Health Organisation classification and grading for neuroendocrine neoplasms (NENs) of the GI tract and hepatopancreatobiliary organs

Terminology	Differentiation	Grade	Mitotic rate^*^	Ki-67%
			(mitoses/2 mm^2^)	index^*^
NET, G1	Well differentiated	Low	< 2	< 3%
NET, G2	Well differentiated	Intermediate	2–20	3–20%
NET, G3	Well differentiated	High	> 20	> 20%
SCNEC	Poorly differentiated	High^†^	> 20	> 20%
LCNEC	Poorly differentiated	High^†^	> 20	> 20%
MiNEN	Well or poorly differentiated^‡^	Variable^‡^	Variable^‡^	Variable^‡^

*LCNEC* large-cell neuroendocrine carcinoma, *MiNEN* mixed neuroendocrine-non-neuroendocrine neoplasm, *NEC* neuroendocrine carcinoma, *NET* neuroendocrine tumour, *SCNEC* small-cell neuroendocrine carcinoma

*Mitotic rates are to be expressed as the number of mitoses/2 mm^2^ as determined by counting in 50 fields of 0.2 mm^2^ (i.e. in a total area of 10 mm^2^); the Ki-67 proliferation index value is determined by counting at least 500 cells in the regions of highest labelling (hot-spots), which are identified at scanning magnification; the final grade is based on whichever of the two proliferation indexes places the neoplasm in the higher-grade category

†Poorly differentiated NECs are not formally graded, but are considered high grade by definition

‡In most MiNENs, both the neuroendocrine and non-neuroendocrine components are poorly differentiated, and the neuroendocrine component has proliferation indices in the same range as other NECs, but this conceptual category allows for the possibility that one or both components may be well differentiated; when feasible, each component should therefore be graded separately

Gastric neuroendocrine neoplasms (g-NENs) are relatively rare, and multiple observational studies have shown that they account for approximately 7% of all digestive NENs [2] and less than 1% of all gastric neoplasms [3]. However, the incidence of g-NENs has increased in most countries over recent decades, in part because of greater awareness of the disease among clinicians, improved diagnostic techniques and more widespread use of upper gastrointestinal endoscopy [4]. This has led to improvements in our understanding of disease biology, contributed to changes in management and opened new avenues of research. In this article, we will discuss some the most exciting recent developments in understanding of the molecular pathogenesis, management and prognosis of gastric NENs over the last 5 years. We identified the themes and references that have been included in this article by searching PubMed from 2016 to 2021 using the key search terms ‘gastric, stomach, neuroendocrine, carcinoid, neuroendocrine carcinoma, mixed neuroendocrine-non-neuroendocrine neoplasms’.

## Current Classification and Management of Gastric NENs

**Fig. 1:**
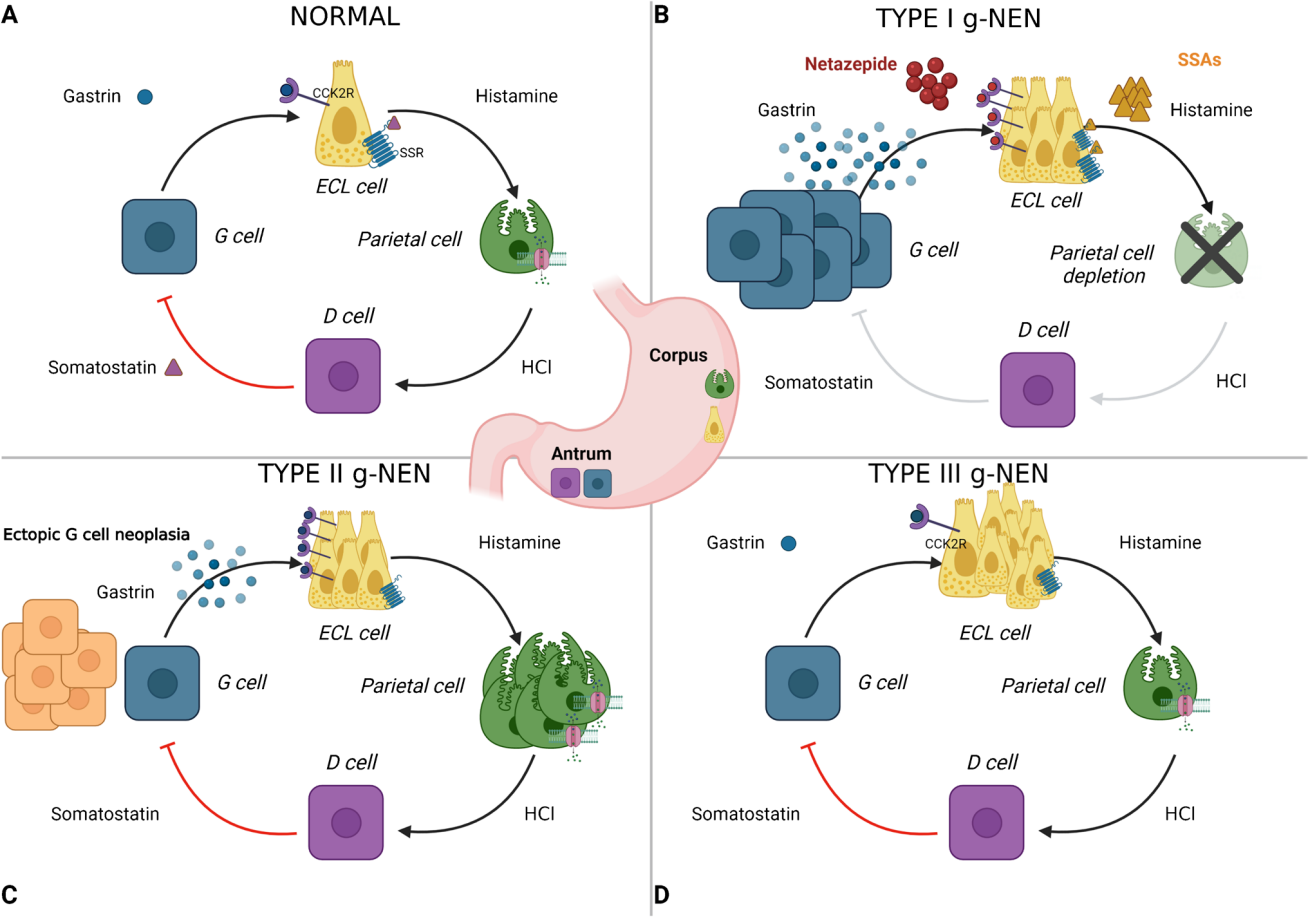
Pathophysiology of g-NENs. **A** Physiological gastric acid secretion: Antral G cells release gastrin after the ingestion of food. Gastrin binds to the CCK2 receptor on corpus-located enterochromaffin-like (ECL) cells, and this cell type then produces histamine. Histamine then binds to H2 receptors on gastric parietal cells resulting in stimulation of acid production and a reduction in the pH in the gastric lumen. This initiates an inhibitory feedback loop to downregulate gastrin release mediated by somatostatin-producing D cells which directly inhibits further release of gastrin from G cells. **B** Type I g-NENs: Loss of parietal cells disrupts the inhibitory loop leading to antral G cell hyperplasia and hypergastrinaemia. This leads to ECL-cell hyperplasia and eventually type I g-NEN formation. Potential treatment targets on the ECL cell are the CCK2 receptor and the somatostatin receptor (SSR). Netazepide irreversibly binds to the CCK2 receptor, and somatostatin analogues (SSAs) bind to the SSR with anti-proliferative effects. **C** Type II g-NENs: Ectopic G cell neoplasia leads to hypergastrinaemia and resultant excess gastric acid production that is independent of the normal inhibitory feedback loop. ECL cell and parietal cell hyperplasia are observed. **D** Type III g-NENs: Sporadic formation of g-NEN derived from ECL cells occurs with no evidence of hypergastrinaemia and no known disturbance of underlying gastric physiology. Created with BioRender.com

Gastric NENs are thought to arise from subepithelial, histamine-secreting, enterochromaffin-like (ECL) cells and are conventionally subdivided into three main types which have very different and distinct pathophysiologies (Fig. 1). Type I g-NENs are associated with autoimmune atrophic gastritis and hypochlorhydria, while type II g-NENs develop in some patients who have gastrinomas, increased gastric acid secretion, Zollinger-Ellison syndrome (ZES) and multiple neuroendocrine neoplasia (MEN) type 1. Both type I and type II g-NENs are associated with elevated fasting serum gastrin concentrations, and many patients have a relatively indolent disease course and good prognosis. Hypergastrinaemia exerts a proliferative effect on ECL-cells in the stomach, leading to linear and micronodular ECL-cell hyperplasia and subsequently dysplasia and NEN development. By contrast, type III g-NENs are sporadic tumours, and gastrin is not thought to be involved in their pathogenesis. Type III g-NENs tend to behave more aggressively and often have a poorer prognosis.

In addition to these classical three subtypes, the existence of a type IV g-NEN has been suggested. One proposition has been that this fourth type has similar characteristics to type III g-NENs, but an even more aggressive nature. This type of g-NEN may arise from other types of endocrine cells (that secrete serotonin, gastrin or adrenocorticotrophic hormone) and be a poorly differentiated NEC [5, 6]. In modern NEN classification systems, however, it is likely that this type of type IV g-NEN simply represents a type III g-NEN which has features of a NEC rather than a NET. A second different proposal for a type IV g-NEN involves a g-NEN that has developed in a patient who has an intrinsic defect in the secretion of acid by gastric parietal cells. This type of type-IV g-NEN is characterised by hypochlorhydria, hypergastrinaemia and multiple small gastric polyps (similar to type I), but histological examination of the gastric corpus mucosa classically reveals hypertrophy and hyperplasia of parietal cells, with a vacuolated cytoplasm suggesting a structural abnormality that prevents acid secretion [7–9]. This type of g-NEN is very rare, and the mechanism of pathogenesis is similar to type I, so it is not frequently included as a separate entity in g-NEN classification systems.

Clinically, most g-NENs tend to be asymptomatic, and many tumours are identified incidentally during an endoscopy that is being performed to investigate unrelated symptoms or anaemia. Once detected, it is essential to determine the subtype of g-NEN through biochemical, histological and endoscopic assessment in order to provide appropriate management. The three main subtypes of g-NEN have typical clinical and biochemical features (Table 2). Computerised tomography (CT) with gastric distention can be helpful for tumour staging and provides information that is complementary to gastroscopy and histological evaluation. In contrast to well differentiated g-NETs, g-NECs or g-MiNENs are more likely to be found in the gastric fundus and cardia, have larger size, grow infiltratively, have unclear tumour margins, involve the serosa, appear ulcerated, demonstrate heterogeneous enhancement and have metastatic lymph nodes (LN) detected on CT scan [10]. The appearance of intact overlying mucosa with mucosal tenting can also sometimes help in discriminating between g-NECs and gastric adenocarcinomas [11]. Furthermore, a larger size and greater necrotic volume of metastatic LNs can also be a feature of g-NECs rather than gastric adenocarcinoma [11].

**Table 2:** Types of gastric neuroendocrine tumours, general characteristics in endoscopic appearance, histology and management

	Type I	Type II	Type III
Proportion, %	70–80	5–10	15–20
Gastric localisation	Corpus, fundus	Body, fundus, antrum	Antrum or corpus
Typical endoscopic and morphological characteristics	Single/multiple (60%), small (< 1 cm); polypoid or submucosal	Often multiple, small (< 1–2 cm); polypoid (sessile)	Single, large size (> 2 cm); occasionally ulcerated
Associated disorders	Chronic atrophic gastritis and pernicious anaemia; achlorhydria	Gastrinoma/multiple endocrine neoplasia-1	Sporadic
Histology	Well differentiated	Well differentiated	Well differentiated, poorly differentiated or mixed endo/exocrine
	(G1–G2)	(G1–G2)	(G1,2,3 NET or NEC)
Fasting serum gastrin levels	↑	↑	Normal
Gastric pH	↑↑	↓	Normal
Investigations	•Endoscopic assessment: number, size and location of tumour(s), tumour biopsies, assess background gastric mucosa, biopsies of gastric antrum and corpus, pH of gastric juice		
	• Biochemical assessment: fasting plasma gastrin and chromogranin A, anti-gastric parietal cell and intrinsic factor antibodies, thyroid function tests, FBC, vitamin B12		
	• Histological assessment: Ki67% and mitotic index, LVI, grade. Gastric corpus: atrophic gastritis, intestinal metaplasia, ECL cell hyperplasia. Antrum: G cell hyperplasia and *H. pylori* infection		
	• Endoscopic ultrasound scan (EUS)		
	• CT/MRI scan		
	• Somatostatin Receptor Imaging		
Management	*Tumours < 10 mm*	Treatment of gastrinoma and MEN-1	Partial or total gastrectomy with LN dissection
	Endoscopic surveillance every 1–2 years *Tumours > 10 mm*		
			Systemic therapy for metastatic disease (chemotherapy, SSAs, PPRT)
	No LN involvement and confined to submucosa/lamina propria:		
	Endoscopic resection		
	LN involvement and/or positive margin on endoscopic resection:		
	Surgery (wedge resection, subtotal/total gastrectomy)		
Risk of metastases, %	2–5	10–30	50–100
Prognosis	Excellent	Very good	Poor

†Adapted from ENETS Consensus Guidelines^6^

The management of a g-NEN critically depends on three key parameters: the tumour subtype, histological grade and tumour stage [12]. Treatment options range through endoscopic surveillance with or without endoscopic polypectomy, to medical management, to aggressive surgery and to oncological therapies. Optimal patient management should be determined by a suitably qualified multidisciplinary team of clinicians and is influenced by the type, size, multiplicity, grade and stage of g-NEN. These features have been summarised in Table 2.

## Recent Advances in Understanding the Pathogenesis of Gastric NENs

### Molecular Profiling of Gastric Neuroendocrine Tumours

The rarity of neuroendocrine malignancies limits the biomedical scientific community’s ability to develop new therapies; hence, a better understanding of their underlying tumour biology is critical. A number of recent studies have investigated the molecular features that are associated with gastric NETs, NECs, MiNENs and gastric adenocarcinomas in order to determine the similarities and differences between these tumour types and provide insights into oncogenesis, as well as potential therapeutic strategies. Mutations in MEN1, DAXX and ATRX genes are entity-defining for well-differentiated NETs at other sites, whereas NECs often have TP53 or RB1 mutations [13••]. In some cases, the presence of these mutations may help in diagnosis and determining treatment [14].

Regarding gastric NETs, a recent whole-exome sequencing study analysed a family in which 5 of 10 siblings developed type I g-NENs. A germline mutation in the ATP4a gene was found to be associated with the presence of disease, but mutations of this gene were not detected in a validation cohort of 14 patients who had sporadic type I g-NENs [15]. Two other small studies have also investigated the molecular profiles of type I g-NENs, but they only analysed 22 cases in total [16, 17]. Sporadic mutations were found in less than 15% of cases for the genes tested (TP53 14%; RB1 9%; and SMAD4 9%), with mutations in NET-related genes also being rare (MEN1 7%, ATRX 7%, TSC2 7% and PETN 5%). By contrast, a whole-exome sequencing study of gastric NECs found that they exhibited a specific mutational pattern, with a higher mutational burden compared with gastric adenocarcinoma [18], and the most frequently mutated gene was TP53 (which was affected in 53–100% of the cases examined).

The pathogenesis and genetic features of gastric MiNENs remains poorly understood. Furthermore, while the survival of patients who have gastric adenocarcinomas is improving due to the development of targeted novel therapies, there are currently no targeted or tailored therapeutic protocols for gastric MiNENs. Current hypotheses suggest that the adenocarcinoma and neuroendocrine components of MiNENs have a clonal origin [19]. In support of this, Koh et al. recently demonstrated that the majority of somatic mutations, especially pathogenic mutations of TP53, were identically shared by the two separate components of gastric MiNENs, thus providing further genetic evidence for a common ancestor model for these tumours [20]. A further study that compared NECs and MiNENs also identified TP53 as the single cancer-related gene that was most frequently mutated in both tumour types [21].

### Type I g-NENs and ATP4a Mutations

Calvete et al. used whole exome sequencing to identify a missense mutation (p.R703C) in the human ATP4a gene [15] as the cause of aggressive familial type I g-NENs in homozygous individuals from a single family. Affected individuals showed tumour development at an early age, gastric hypoacidity, hypergastrinaemia, iron-deficiency anaemia, gastric intestinal metaplasia and frequent LN metastasis, with in one case contemporaneous gastric adenocarcinoma.

Traditionally, autoimmune atrophic gastritis in humans develops as the consequence of an IL-17-dependent innate autoimmune response mediated by CD4+ T cells. These T cells recognise the proton pump H^+^/K^+^-ATPase, which is coded by the ATP4a gene on parietal cells [22]. Subsequent activation of the NLRP3 inflammasome-reactive oxygen species pathway results in the destruction of parietal cells [23]. Infection with *H. pylori* also mimics parietal cell antigens in human autoimmune gastritis, promoting destabilisation of the mitochondria, preventing the adaptive immune response and reducing gastric acidity [24].

The functional significance of this newly identified p.R703C mutation in ATP4a has now been investigated further by creating a knockin mouse model [25]. Homozygous mice recapitulated most of the phenotypic alterations observed in human individuals, strongly suggesting that this mutation is the primary alteration responsible for disease development. The mouse model also suggested that achlorhydria might contribute to tumorigenesis to a greater extent than hypergastrinaemia, as gastric acidification prevented or partially reverted the histological changes and rescued abnormal biochemical parameters. Furthermore, the ATP4a p.R703C mutation was shown to deregulate the acid-base balance within parietal cells, affecting mitochondrial biogenesis in a similar manner to *H. pylor*i infection [26••]. Mitochondrial malfunction activated reactive oxygen species signalling, which in turn triggered caspase-3-mediated apoptosis of parietal cells; this malfunction was also restored when mice were treated with acidified water.

Genetically mediated ATP4a mutation induced gastric atrophy and *H. pylori* induced gastric atrophy therefore seem to alter mitochondrial function through similar mechanisms, but these are different from those found in classical autoimmune atrophic gastritis. Importantly, mitochondrial function in the mouse model was partially recovered when euchlorhydria/gastric acidity was restored during prevention and reversion experiments. This suggests that restoration of gastric acidity might be helpful in such patients and might even have a role in limiting the progression to *H. pylori* induced atrophic gastritis.

## Development of Potential Novel g-NEN Biomarkers

Biomarkers are important adjuncts in the treatment and follow-up of patients with many diseases. For example, the measurement of secretory markers such as gastrin, insulin, glucagon and vasoactive intestinal peptide can be very helpful in monitoring the success of therapy in patients who have specific functional pancreatic NETs [27]. However, a clinically useful universal g-NEN biomarker which identifies both functional and non-functional tumours, which correlates with disease aggressiveness or progress and which is useful for diagnosis and surveillance does not currently exist. Currently employed serum g-NEN biomarkers such as gastrin and chromogranin A concentrations are unfortunately largely ineffective [28]. For example, elevated fasting serum gastrin concentrations are non-specific, and hypergastrinaemia can result from chronic atrophic gastritis, Zollinger-Ellison syndrome, persistent *Helicobacter pylori* infection and patients taking proton pump inhibitors [29]. The combination of low pepsinogen I/II ratio with high gastrin levels has therefore been proposed as more specific method to characterise and risk stratify patients who have autoimmune atrophic gastritis and type I g-NENs [30]. Measurement of serum chromogranin A concentration also has significant clinical limitations, particularly its lack of sensitivity in determining tumour stage and location, but the results can also be influenced by the specific assay that is used, limiting direct comparison of results that have been obtained at different laboratories [31]. These limitations have led to interest in developing novel biomarkers for g-NENs. This includes the development of potential novel immunohistochemical biomarkers, as these have been shown to provide valuable prognostic and predictive information in other tumour types [32].

### Circulating Biomarkers

#### Circulating Tumour mRNA

An emerging molecular liquid biopsy method involving circulating tumour RNA, the NETest, has been recently developed for several types of NEN. This innovative approach involves the simultaneous measurement of 51 different NET-related transcripts. These cover a multiverse of neuroendocrine cell and tumour biology, and the test yields a single readout through an undisclosed algorithm [33]. In a recent study by Malczewska et al. involving 46 g-NENs (42 type I and 4 type III, of which 3 were NECs), the NETest was positive in patients who had both type I and type 3 g-NENs [34•] with a sensitivity of 100% , specificity of 87% and overall accuracy of 90%. Elevated test results were found in all patients with who had evidence of macroscopic and microscopic residual disease as well as in some patients who had no macroscopic or microscopic disease (possibly as a consequence of background ECL-cell hyperplasia). Therefore, this sensitive liquid biopsy test has potential utility in the future management and surveillance of g-NENs. Further research studies to determine the usefulness of this test in clinical practice and during follow-up are however needed prior to its routine implementation.

#### miRNAs

MicroRNAs (miRNAs) represent potential novel biomarkers for g-NENs. miRNAs are a class of endogenous non-protein coding short RNAs that post-transcriptionally regulate approximately 30% of the human genome [35, 36]. As miRNAs control a large proportion of the genome, their expression patterns are tissue-specific, and dysregulation has been observed in many malignancies [37]. This suggests that miRNAs may have a role as biomarkers that may prove useful in determining cancer diagnosis, prognosis and response to therapies. miR-222 expression has been found to be increased in both the stomach and serum of patients who had type I g-NENs as well as hypergastrinaemic patients who had autoimmune atrophic gastritis [38]. Furthermore, treatment with the gastrin/CCK2 receptor antagonist, netazepide, was shown to reduce miR-222 abundance. miR-222 therefore has potential utility as a biomarker for response to gastrin/CCK2R antagonist therapy in g-NETs, but further studies involving larger numbers of patients are again needed prior to implementation of this test into routine clinical practice.

### Immunohistochemical Biomarkers

#### PDL1-MMR-MSI

Immunotherapy with immune checkpoint inhibitors (ICIs) has recently been shown to be effective in several metastatic cancers, including malignant melanoma, non-small lung cancer and renal cell carcinoma [39]. Programmed cell death ligand 1 (PD-L1) and programmed cell death protein 1 (PD-1) are two immune checkpoint proteins that play a major role in cancer immunity [40], and these proteins are the targets of some ICI therapies. In some situations, expression of PD-1 has therefore been shown to help in predicting clinical response to ICIs. Furthermore, microsatellite instability (MSI), which is typically caused by mutations in mismatch repair (MMR) genes including MLH1, MSH2, MSH6 and PMS2, has also emerged as a robust and consistent pan-tumour biomarker that predicts benefit in patients treated with ICIs [41].

MSI has been proposed to promote tumour development in both NECs and MiNENs [42–44]. Gastric MSI-NECs and MiNENs have been shown to resemble MSI-gastrointestinal adenocarcinomas in terms of their frequency, molecular profiles and pathogenetic mechanisms [42] and thus potentially represent a subset of patients who may be more likely to respond to ICI therapy. A recent study by Yamashita et al. [45•] also identified high PD-L1 expression in 72% of cases in a cohort of 25 g-NEC/MiNEN patients, while Yang et al. found high PD-L1 expression in 49% of 43 g-NECs, and this correlated with poorer prognosis [46]. Whether these observations have implications for the clinical assessment of MSI and PD-L1 status in g-NECs/MiNENs and whether the findings then represent biomarkers that can used to predict response to ICI therapy and influence prognosis remain to be established. However, this is a potentially exciting area of research that may lead to personalisation of treatment approaches and certainly warrants further evaluation.

#### ALDH1A1

High ALDH1A1 expression may also be a prognostic indicator in patients who have g-NECs. The aldehyde dehydrogenase (ALDH) family of proteins are present at high levels in stem cells as well as cancer stem cells in a number of malignancies [47–49] as well as NETs [50]. In gastric adenocarcinoma, overexpression of ALDH1A1 is significantly associated with depth of tumour invasion and LN metastases [51] and predicts poor prognosis in terms of overall survival and recurrence free survival. Ye et al. recently examined the expression of ALDH1A1 in g-NECs and found similar results [52]. High ALDH1A1 expression levels were associated with LN status, lymphovascular invasion (LVI) and Ki-67 index. Furthermore, strongly positive ALDH1A1 expression was found to be an independent prognostic factor associated with poorer survival rates.

## Recent Developments in g-NEN Patient Management

### Type I g-NENs

Current European Neuroendocrine Tumour Society (ENETS) guidelines recommend conservative management for most type I g-NENs [53]. For this tumour type, the risk of metastatic potential is directly correlated to tumour size [54]. Most patients who have small tumours are therefore enrolled onto endoscopic surveillance programmes, where tumour number and size are monitored and regular histological assessment is undertaken. Endoscopic surveillance has also been advocated by a number of organisations for all patients who have atrophic gastritis, as previous studies have reported a varying progression rate to gastric adenocarcinoma up to 2% per year during long-term follow-up [55, 56]. Current guidelines therefore suggest annual or 2-yearly endoscopic surveillance of type I g-NENs with biopsy sampling of polyps, and endoscopic resection is advised for lesions measuring more than 10 mm in diameter [53].

Recent analysis of the outcomes of endoscopic surveillance programmes [57, 58] has indicated that about 30% of type I g-NET patients will require intervention in the form of endoscopic resection or surgery at some point during their disease course. A reintervention rate of at least 50% (7/13 patients and 44/84 patients in each study) was noted after a median follow-up of 22 and 11 months after first intervention, with a further 50% of patients experiencing multiple recurrences. Interestingly, both of these studies reported that high fasting serum gastrin concentrations were more likely to be associated with disease progression. Multiple endoscopic techniques have been used to resect type I g-NENs including band and slough technique [59], endoscopic mucosal resection (EMR) [60] and endoscopic submucosal dissection (ESD) [61]. All these techniques are feasible and effective with a good safety profile, but no published research to date has suggested that one technique seems to be more effective than the others [62] .

Netazepide is a gastrin/CCK2 receptor antagonist which has recently been identified as a potential novel treatment for hypergastrinaemia-associated conditions [29], with evidence from non-clinical models as well as clinical studies in healthy volunteers [63, 64]. Furthermore, due to the rarity of g-NENS, it has been designated an orphan medicinal product (with dedicated funding) in Europe and the USA for the treatment of type I g-NENs [65]. Treatment with netazepide over 12 weeks was found to reduce the number and size of type I g-NETS, plasma chromogranin A levels and gastric mucosal biomarkers including chromogranin A [66, 67]. This treatment also resulted in similar responses and had a good long-term safety profile when administered to the same patients over a subsequent 52-week period [68]. Further work investigating the action of netazepide using gene expression profiling noted a reduction in the expression of PAPPA2, a metalloproteinase that increases the bioavailability of insulin-like growth factor (IGF) [69•]. These findings were confirmed using patient biopsies acquired pre and post netazepide treatment and suggest that a novel gastrin-dependent IGF signalling pathway may be important in the development of type I g-NETs. Netazepide is therefore possibly an alternative systemic management strategy for some type I g-NETs, either alone or in combination with endoscopic surveillance, although additional larger clinical trials are needed to confirm these initial findings

An alternative medical management option that has recently been evaluated in type I g-NETs involves the use of long-acting somatostatin analogues (SSAs), as these inhibit gastrin release and thus inhibit endocrine cell proliferation [70, 71]. In a recent meta-analysis of results from selected patients who could not be safely managed by endoscopic follow-up or resection due to multiple or frequently recurring disease, SSAs were shown to induce an excellent response rate (i.e. a cumulative complete response rate of 84.5% when considering six prospective studies) after 12 months of therapy. This treatment also had a good safety profile, but was associated with a high cumulative relapse rate of 30% during 34 months of follow-up [72•].

Surgery, in the form of an antrectomy, has historically been advocated to treat some type I g-NENs. This intervention leads to a reduction in circulating gastrin concentrations by removing the anatomical source of hypergastrinaemia. However, it is not effective in all patients, and it carries the risk of morbidity and mortality [73, 74]. No significant recent advances in the role of surgery in the management of type I g-NETs were identified during the literature search that was undertaken in preparation for this article.

### Type III g-NENs

For localised type III g-NENs, due to their potentially more aggressive behaviour, surgical resection, in the form of a partial or total gastrectomy with LN dissection, remains the recommended treatment according to the ENETS consensus guidelines [53]. However, a recent systematic review suggested that the majority of type III g-NENs diagnosed in the modern era have a lower grade than historically reported [75•]. Criteria for selecting patients for endoscopic or local resection (without formal LN dissection) have now been accepted for managing other tumour types, including early gastric adenocarcinomas using endoscopic mucosal resection or laparoscopic wedge resection [76, 77] and selected NENs at other sites such as the appendix (by simple appendectomy) [78] and rectum (through transanal excision) [79]. Similar criteria have recently been proposed for type III g-NENs, by defining those tumour subgroups which are associated with a lower rate of LN metastasis. Three recent studies from Asia [80–82] and one from Europe [83•] all concluded that local excision in the form of surgical wedge resection or endoscopic resection is a suitable treatment option for certain type III g-NENs when the following criteria are satisfied: well differentiated histology, low grade (grade 1 and potentially some grade 2 NETs with Ki67 index < 10%), tumour size less than 10 (or possibly 15) mm, depth of invasion confined to the submucosal layer and no evidence of LVI.

## Recent Developments in g-NEN Patient Outcomes and Prognostic Indicators

Predicting the outcome of patients with g-NENs is complicated, as these tumours are biologically very heterogeneous, so prognosis and management are affected by a number of factors. Multiple studies have demonstrated that patient age, tumour grade, size and type, gender and marital status are prognostic factors that influence clinical outcome in g-NENs [84–86]. Perineural invasion in high grade g-NENs is also a predictor of outcome [87]. Neutrophil to lymphocyte ratio in a preoperative blood sample has also been identified as an independent prognostic factor of recurrence free survival and overall survival after surgery [88]. In resected g-NECs, metastatic LN number more than two, the presence of metastatic disease in more than 10% of resected LNs and involvement of station 2 LNs have all been shown to be significant prognostic indicators associated with a poorer prognosis [89]. These features have recently been incorporated into nomograms which can be used to estimate the survival of individual g-NET and g-NEC patients [90]. However, to date, these nomograms have not accounted for the different NET subtypes; thus, additional research in this area is warranted.

The mainstay of treatment of non-metastatic MiNENs and the only chance of cure remains surgical resection. If an adjuvant treatment is indicated, or if the neoplasm is not resectable, the type of chemotherapy administered is dependent on the histological characteristics of each component malignancy (including grade and differentiation status) and which component is thought to represent the more dominant tumour type [43]. A recent study compared long-term survival and patterns of recurrence among g-NECs, g-MiNENs and gastric adenocarcinomas [91]. Patients with a g-NEC or g-MiNEN had poorer prognoses than those with a gastric adenocarcinoma. The presence of a g-NEC or g-MiNEN was independently associated with poorer disease-free survival and post recurrence free survival as well as an increased likelihood of developing distant tumour recurrence.

## Conclusions

The heterogeneity and rarity of gastric neuroendocrine neoplasms pose challenges in the understanding and management of this disease. Despite recent advances in the molecular profiling of cancers in general, due to the fragmentary nature of research and the low incidence of g-NENs, no key pathways have yet been identified that are responsible for the development of these tumours. This is currently limiting attempts to molecularly subclassify these tumours and is also hampering efforts to develop novel and/or personalised therapeutic strategies. More basic science research is therefore needed to investigate the molecular subtypes and tumour pathogenesis. Preclinical models would be invaluable to provide better understanding and insight into treatment options. However, the usefulness of currently available neuroendocrine neoplasm cell lines is questionable [92], none have been derived from the stomach, and there are also limited animal models available [93]. Recently, promising NEC organoid models have been established [94], and such technology may also in future be suitable for modelling g-NETs. Recent clinical studies have demonstrated that less aggressive treatment options such as medical management or endoscopic treatment may be appropriate in patients who have smaller and more indolent g-NETs. However, larger clinical studies are still needed to better define eligibility for these treatment strategies as well as to improve patient outcomes in g-NECs and g-MiNENs.
